# Model of Early Stage Intermediate in Respect to Its Final Structure

**DOI:** 10.3390/biom9120866

**Published:** 2019-12-12

**Authors:** Piotr Fabian, Katarzyna Stapor, Irena Roterman

**Affiliations:** 1 Institute of Computer Science, Silesian University of Technology, Akademicka 16, 44-100 Gliwice, Poland; piotr.fabian@polsl.pl (P.F.); katarzyna.stapor@polsl.pl (K.S.); 2 Department of Bioinformatics and Telemedicine, Jagiellonian University-Medical College, Łazarza 16, 31-530 Kraków, Poland

**Keywords:** protein folding, early stage, hydrophobicity, structural codes

## Abstract

The model, describing a method of determining the structure of an early intermediate in the process of protein folding to analyze nonredundant PDB protein bases, allows determining the relationship between the sequence of tetrapeptides and their structural forms expressed by structural codes. The contingency table expressing such a relationship can be used to predict the structure of polypeptides by proposing a structural form with a precision limited to the structural code. However, by analyzing structural forms in native forms of proteins based on the fuzzy oil drop model, one can also determine the status of polypeptide chain fragments with respect to the assumptions of this model. Whether the probability distributions for both compliant and noncompliant forms were similar or whether the tetrapeptide sequences showed some differences at a level of a set of structural codes was investigated. The analysis presented here indicated that some sequences in both forms revealed differences in probability distributions expressed as a negative statistically significant correlation coefficient. This meant that the identified sections (tetrapeptides) took different forms against the fuzzy oil drop model. It may suggest that the information of the final status with respect to hydrophobic core formation is already carried by the structure of the early-stage intermediate.

## 1. Introduction

The protein folding process is seen as a multistage process [1,2,3,4,5,6,7,8]. For homologous proteins the methods predicting the structure of proteins are based on the so-called comparative modeling (Darwinian model) [9]. Using this technique, a starting structure for the energy minimization procedure is constructed based on the similarity of homologous proteins [9,10,11,12]. If individual fragments take forms derived from a larger number of proteins, the used method of genetic algorithms allows for combining these fragments into one whole [13]. Other techniques—based on the so-called ab initio model—tend to obtain an initial starting structure for the energy minimization procedure using the so-called coarse-grained model [14,15,16,17]. The introduction of this model dramatically reduces the number of degrees of freedom and makes it easier to search the conformational space in a much shorter computational time. In the Rosetta system, a starting structure is determined based on the frequency of occurrence of given conformations for specific tripeptides and nine peptides. The large number of proposals obtained in this way is subjected to a clustering procedure leading to a reduction in the number of candidates [18,19,20].

Programs based on homology modeling and gluing of short peptide structures are among the best in terms of the CASP (Critical Assessment of Structure Prediction) competition criteria [21,22].

The model discussed in this work also refers to a database of known structures that are analyzed based on the so-called early intermediate model. It is based solely on the backbone structure, using only the description of the chain geometry expressed by the radius of curvature, which is a result of the angle of aperture between adjacent planes of peptide bonds resulting from the rotation angles of Phi and Psi [23,24,25].

The late intermediate structure—the fuzzy oil drop model—assumes that the spatial structure of proteins is obtained by the micellization mechanism [26]. So far, the presence of hydrophobicity distributions consistent with a three-dimensional (3D) Gaussian distribution has been shown, that is, the maximum concentration of hydrophobicity occurs in the center and decreases, as it moves away from the center until reaching a level of close to zero on a protein surface [27]. Such distributions were observed in globular proteins [28] and multidomain protein [29]. A set of amino acids in a polypeptide chain sequence enables or excludes unicentric distribution. The inability to obtain decomposition in accordance with an idealized spherical micelle is expressed by the presence of a local excess of hydrophobicity on a surface and a local deficit. In the first case, such exposure is used to complex another protein [30]. In the second case, the deficit is identified in the presence of a ligand-binding cavity [31]. A specific example of noncompliance of a hydrophobicity distribution with a 3D Gaussian distribution is amyloids, where the hydrophobicity distribution corresponds to the distribution present in the case of a band micelle. The diversity of hydrophobicity density takes the form of bands extending along the long axis of an amyloid fibril in these cases [32].

The protein folding model reflecting the folding process assumes the presence of two stages: early and late. The structure of the early intermediary is constructed based on a structural alphabet described in detail in [25]. The alphabet consists of seven codes (A–G) corresponding to appropriately highlighted areas of a Ramachandran map [25] determined on the basis of the probability distribution of the respective angles Phi and Psi.

3D protein structures available in the PDB (Protein Data Bank) (nonredundant set) were identified using structural codes. Structural identifiers are given in sets for tetrapeptides, which results in a contingency table with 160,000 columns (number of sequence combinations for 20 amino acids) and 2401 rows for structural codes (number of combinations for seven structural codes) [33]. Two contingency tables were constructed for structures compatible with a spherical micelle system and for fragments with a distribution incompatible with a micellar distribution. A comparison of the frequencies of observation of structural codes occurring for selected sequences—tetrapeptides as assumed—should answer the question whether a decision on single-center ordering is made already at the stage of an early intermediate. A negative correlation coefficient for the frequency of occurrence of a given combination of structural codes for a given tetrapeptide sequence is expected to indicate these structural forms that differ in areas compliant with micellar distributions versus those with noncompliant distributions. Similarly, a negative correlation coefficient for selected sequences (tetrapeptides) may indicate that these sequences more significantly differentiate structural forms in the early intermediate in the micellar distribution system (spherical micelle) compared to those in the system deviating from the system of the spherical micelle.

## 2. Materials and Methods

### 2.1. Generation of a Nonredundant Protein Set

The nonredundant protein set was created on the basis of a regularly (weekly) updated set of protein clusters from the entire PDB database [34,35].

Clustering of all proteins was done using the BlastClust program (https://www.ncbi.nlm.nih.gov/Web/Newsltr/Spring04/blastlab.html) [36] in such a way that a single cluster contains proteins similar to each other whereas proteins in different clusters are extremely dissimilar. The number of clusters obtained depends on the parameters of the clustering algorithm used. A set of clusters with a 95% similarity was chosen. To create a nonredundant protein set, one representative was selected from each cluster.

Due to the fact that in further analysis (i.e., testing compliance with the fuzzy oil drop model) we did not consider the entire protein from the PDB, but only a single domain, each protein from the above-defined nonredundant set was divided into domains based on information from the CATH database [37,38]. The resulting set had 68,942 domains. Domains were assumed to be structural units according to the definition, that is, a domain is a structural form that folds as an individual structural unit. In a large protein molecule, the incompatibility status of a particular amino acid or chain fragment may result from inter-domain relationships. For example, the gap between domains may be the result of inter-domain interactions, with domains formed as independent entities and an inter-domain system resulting from the interactions of already shaped domains. Therefore, this effect that occurs in the subsequent stages of shaping the final protein structure is not necessarily a result of the presence of a centric nucleus in a multidomain protein and was not taken into account.

### 2.2. Identification of Structural Codes

In order to validate the model, a Ramachandran chart was generated on a new created current-domain database. Next, according to the definition of the structural code proposed in [25], each point (Phi, Psi) on the Ramachandran chart was projected onto an ellipse and presented as the angle t corresponding to this point of the ellipse in its parametric equation.

**Figure 1:**
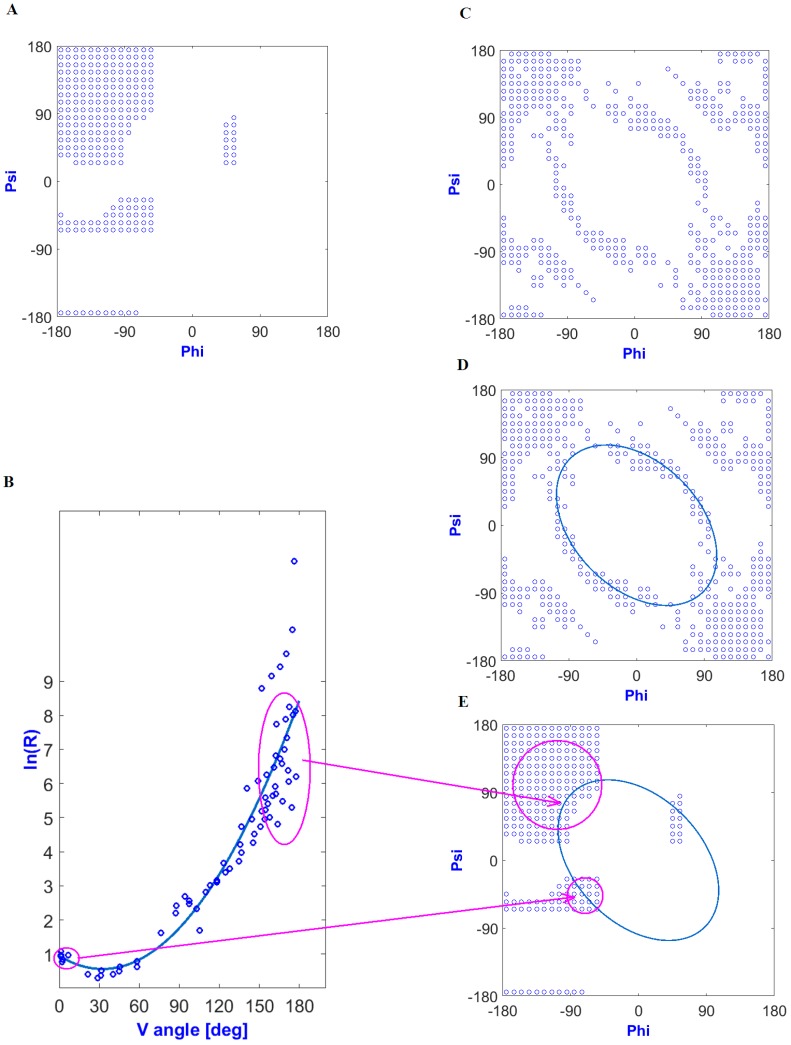
Determination of early intermediate structural codes: (**A**) low-energy part of a Ramachandran plot; (**B**) relationship between the radius of a curvature (In(R)) and the angle of an aperture between adjacent peptide bond planes (dihedral angle) (V angle). Structures fulfilling the condition in the form of an idealized relationship (according to the regression function) located on the Ramachandran map form an elliptical path; (**C**) locations of structures meeting the conditions resulting from the approximation function—relaxed structural forms; (**D**) determination of the elliptical path by approximation; (**E**) relation of a secondary structure to the elliptic curve.

The elliptical path was defined as a result of the dependence of the five-peptide curvature radius on the angle of aperture between adjacent peptide bond planes. This relationship had the form of a second-degree polynomial function (Figure 1B) for low-energy structures (Figure 1A). Structures showing an ideal solution (the radius of a curvature for a given angle of an aperture by potential function) were arranged on the Ramachandran map in the form of an elliptical path (Figure 1C,D). Interpretation of the ln(R) as dependent on V angles in the context of secondary structures is shown in Figure 1E. Assuming that the idealized relationship expressed a relaxed form (resulting only from the specificity of a peptide bond without distorting interatomic interactions), the set of angles Phi_e_ and Psi_e_ (the index “e” means elliptical and thus belongs to the elliptical path), relying on Phi and Psi values of angles observed in a given structure projected onto the ellipse (the shortest distance criterion), determined the conformation treated as a primary structure resulting solely from the specificity of peptide bonds. Therefore, the set of Phi_e_ and Psi_e_ angles was treated as a starting conformation referred to as “early stage”.

The first stage (early stage) of the folding process therefore assumes decision-making power only on the side of backbone preferences. The second stage of the folding model is to introduce inter-atomic interactions. This results in conformational changes dictated by low-energy interatomic (inter-residual) systems. This, of course, entails a change from the Phi_e_ and Psi_e_ angles to the Phi and Psi values, respectively, which are present in the final (native) structural form of proteins.

**Figure 2:**
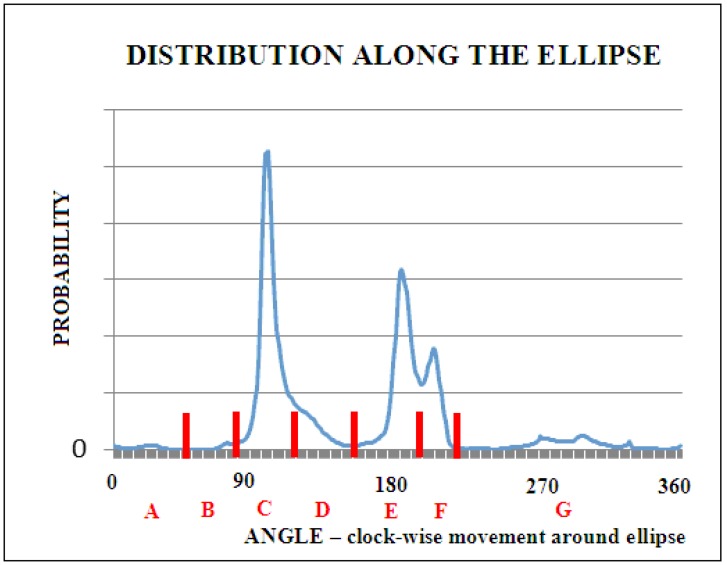
The probability distribution of the Phi_e_ and Psi_e_ angles. The blue line represents the histogram of the number of occurrences of individual t angles for intervals of one degree. The vertical red lines denote successive local maxima, of which structural codes are given below (red letters). The angle was calculated clockwise along the ellipse, starting from the point in the lower right quadrant of the Ramachandran map.

The transformation from the Phi and Psi angles (as they are observed in the PDB database) to the Phi_e_ and Psi_e_ angles, respectively, not only reveals a varied distribution of probability density along the elliptical path, showing preferences in the form of dominance of heliac structures (structural code designated as C), but also reveals the inter-amino acid diversity. Seven local maxima on the elliptical path were identified. Each of them was assigned a code from A to G (Figure 2).

Our research included a larger number of domains than previous ones [25]. This made it possible to plot a more accurate histogram of the frequency of t angles. One degree intervals were used, which provided the histogram with 360 points. If the maxima of individual t angle values were visible in the histogram, this was considered as the middle point of the interval. Histograms were also prepared and broken down into individual amino acids. Twenty detailed histograms were created in this way. It turned out that the dependencies of histograms on amino acids were not visible. Therefore, common boundaries of seven intervals for all the amino acids were assumed.

Figure 2 presents a collective histogram for representative proteins from the PDB database, which are not broken down into amino acids.

Based on the above histogram, new common boundaries of seven intervals for all the amino acids corresponding to structural codes A to G were proposed. They are presented in Table 1. The designated contingency table and the zone ranges for the structural codes have changed since the previous analogous analysis [25]. The change results from a much larger database available today compared to the database that was available twenty years ago [25].

### 2.3. Identification of Structures Compliant with a 3D Gaussian Distribution

After obtaining individual domains, they were assessed for compliance with the fuzzy oil drop model [27] based on relative distances (RDs) [27]. RDs assess the degrees of proximity of a hydrophobicity density distribution observed in a protein (called O) (resulting from inter-residual interactions) to an idealized O-T distribution expressed by means of a 3D Gaussian distribution (called T) (centric hydrophobic nucleus) and to an O-R distribution occurring in a protein without any density variation (called R). These distances are measured using the Kullback-Leibler divergence entropy [39]. The parameter RD assesses the O-T distance in relation to the sum of O-T and O-R distances. Therefore, when the RD value was below 0.5, the protein was assessed as compliant with the fuzzy oil drop model. Domains that were thus rated as noncompliant with the model were then modified to become “more compliant” and were included in the set of “compliant domains”. Such modification operation was carried out on domains not compliant with the model by determining the position of a chain that showed the largest incompliance (i.e., the largest difference between theoretical and observed hydrophobicities) with the fuzzy oil drop model and removing it. This process was repeated until an RD value less than 0.5 was obtained for such a shredded domain. Finally, each position of the originally noncompliant protein was assessed as “compliant” or “noncompliant”. Sequences composed of at least four elements with the same rating were included in the “compliant” or “noncompliant” set. This procedure resulted in several times more chains in the set of “compliant” proteins than in the set of “noncompliant” proteins, which resulted in the necessity of scaling in further stages of analysis by a factor equal to the size ratio of the large and small sets.

### 2.4. Calculation of Contingency Tables

Preparation of the new contingency table (as defined in [25]), separately for each of the two sets created as described above based on the new larger set of proteins, required the following steps:

1. Designation of structural codes for entire domains;
2. Generation of sequence–structure pairs by moving a 4-position window along the chain; four amino acid symbols and four structural code symbols were read for each window position.
3. In the case of domains not compliant with the fuzzy oil drop model, the window selection procedure additionally used the assessment of compliance of subsequent chain positions.
4. The corresponding counter in the contingency table was increased for each sequence–structure pair.

### 2.5. Correlation Coefficient

For each column (fixed tetrapeptide sequence) and for each row (fixed set of tetrapeptide structural codes), the correlation coefficient was determined and evaluated for significance. A statistical significance level of 0.05 was adopted, which with large numbers resulted in the adoption of a critical test value of 1.96.

## 3. Results

### 3.1. The Dependence of the Probability Distribution of a Given Tetrapeptide Sequence in a Compatible or Incompatible Form with a Micellar Hydrophobicity Distribution in the Final Structural Form of the Proteins

Sequence distributions were compared for two individual sets of structural codes: proteins compatible with the fuzzy oil drop model and those incompatible with this model. Only positive values of statistically significant correlation coefficients were obtained. This means that specific systems expressed by structural codes occurred in a similar way, both in areas built in protein molecules according to the principle of the spherical micelle and in areas that were recognized as inconsistent with the assumptions.

### 3.2. The Dependence of the Probability Distribution of a Given Set of Tetrapeptide Structural Codes in a Form Compatible or Incompatible with a Micellar Hydrophobicity Distribution in the Final Structural Form of the Proteins

By calculating the correlation coefficient between the frequency distributions of a given set of structural codes for the tetrapeptide sequences in forms compliant and noncompliant with the fuzzy oil drop model, the presence of a positive statistically significant correlation coefficient was demonstrated. However, in this analysis, there were items, of which negative correlation coefficients turned out to be statistically significant. These examples are given in Table 1.

A negative value of the correlation coefficient indicated those sequences, of which the distribution expressing the frequency of appearance of structural codes was the opposite between the structure determined by the fuzzy oil drop model as compliant or noncompliant with the structure of the expected hydrophobic nucleus. A list of tetrapeptide sequences showing this property is given in Table 2.

**Figure 3:**
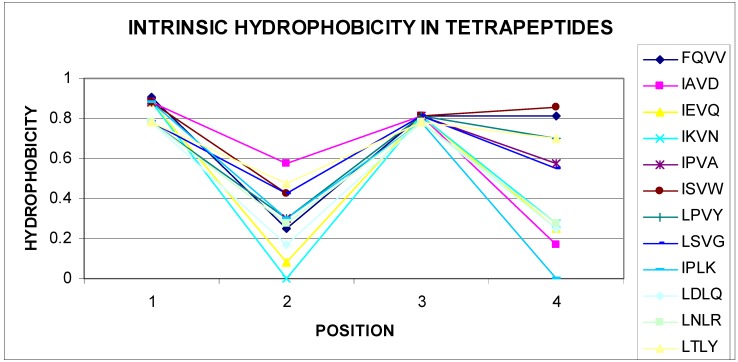
Radically different levels of self-hydrophobicity of amino acids in tetrapeptides (sequences given in the legend).

**Figure 4:**
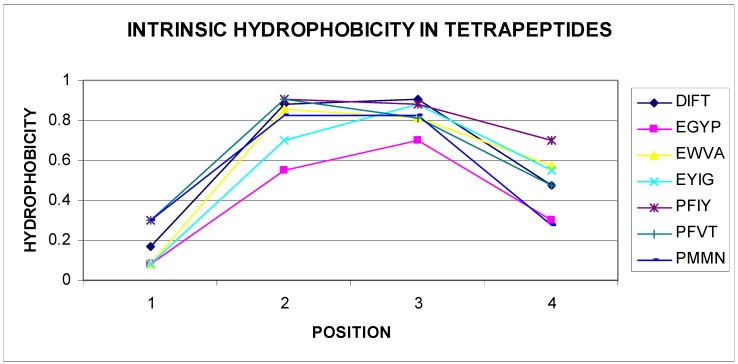
Parabolic change in the self-hydrophobicity of tetrapeptide amino acids. Sequences are given in the legend.

The list given in Table 2 eliminates tetrapeptides, in which the sequence starts with M, R, S, T, V, and W, negative correlation coefficients are not shown. Given the very large set of data (nonredundant PDB database), this conclusion can be generalized. All different tetrapeptide sequences have a number of combinations of 160,000 for tetrapeptides. The number of sequences of tetrapeptides shown in Table 2 is very small, which may indicate high specificity of these sequences. The presence of a negative statistically significant correlation coefficient value for the tetrapeptide sequences mentioned raised the question about the reason for this phenomenon. Gradual changes were observed in structures packed in accordance with the distribution resulting from the 3D Gaussian distribution hydrophobicity in this parameter level. Based on the experience of the authors of this article, areas showing incompatibility with the expected distribution appeared frequently for sequences, in which the change in the level of hydrophobicity (including intrinsic hydrophobicity) changed radically. The analysis of the sequences given in Table 2 confirms these observations. A vast majority of self-hydrophobic distributions in the sequences presented here were characterized by the presence of residues, of which direct neighbors had extreme values. Selected examples are illustrated in Figure 3 and Figure 4.

It should be noted that nonlisted tetrapeptides (with initial amino acids M, R, S, T, V, and W) do not correlate and therefore are not shown in Table 2.

Of course, among the listed sequences are those that seem to be easily adaptable to the environment through gradual, mild changes in the level of hydrophobicity within these tetrapeptides. Examples of a few selected sequences (out of 31 sequences) are shown in Figure 3. The parabolic hydrophobicity system found on the short segment also created unfavorable conditions from the point of view of matching to different forms of a Gauss function, which required a mild change in the level of hydrophobicity when spread on a protein molecule. Centralization of hydrophobic residues for segments of about 10 amino acids required the presence of residues with an elevated level for more amino acids than two on the tetrapeptide segment.

The assessment for the similarity degree of distributions to the status, which was consistent with the nonfuzzy oil drop model for specific sets of structural codes, did not show the presence of a negative statistically significant correlation coefficient. This means that a given set of structural codes—indirectly secondary structure—is not a factor in differentiating against a status that a given tetrapeptide represents in the final structure of the polypeptide.

## 4. Discussion

If the analysis of the relationship between the distributions in the contingency table for compliant and noncompliant forms in the final structure of proteins does show differences, it means that the structure of a given tetrapeptide generated at the stage of an early intermediate does not carry information on its status in the final structure. Indeed, it turns out that only a positive statistically significant correlation coefficient appears for compared sequence distributions with a specific set of structural codes. This means that the structural form—as part of the secondary structure—is independent of the status of a segment (tetrapeptide) in relation to the distribution of hydrophobicity.

In contrast, the relationship between the distributions of the sequence (tetrapeptide) in two forms—compliant and noncompliant with the fuzzy oil drop model—showed, apart from positive statistically significant correlation coefficient values, the presence of differences expressed with negative statistically significant correlation coefficients. This meant that the sequences shown in Table 2 took different structural forms for the early intermediate, either a form compatible with or a form incompatible with the model, assuming a micellar distribution in the final version of the protein structure. This may mean that, already at the early intermediate stage, a form of coding the status that a given tetrapeptide takes in the final form of a given protein was visible. Since local noncompliance is often associated with various forms of biological activity (e.g., ligand binding and protein complexing), it can be speculated that information about biological activity, in which the tetrapeptide is involved, is already encoded at the stage of an early intermediate. It is significant, however, that both a highly represented helical form and a beta structure do not show differentiation in the context of the early and late intermediates.

The fuzzy oil drop model distinguished the status of whole protein molecules as well as chain fragments by representing a hydrophobic distribution consistent with an idealized 3D Gaussian-based function and those that showed local incompatibility with the idealized distribution. The question posed in the present study aimed to identify the diversified status already at the stage of an early intermediate in the process of protein folding. The analysis showed that the synergy that must take place in order to generate a hydrophobic nucleus, of which the structure consisted of relevant chain sections, did not have its mapping in the structure of the early intermediate. Tetrapeptides shown in Table 2 were sections of the chain, which were important for the overall synergy that led to the formation of the complex structure of the bioactive protein form. This was especially visible in the case of proteins undergoing amyloid transformation, where a different model was chosen for shaping mutual relations, leading from the form of a spherical micelle to the form of a linear bands the presence of which is recognized in ribbon-like micelle [26]. This required a change in the organization system of the entire chain and thus the choice of a different synergy system.

The divergence entropy introduced by Solomon Kullback and Richard Leibler [39] played a critical role in the structure analysis. The main advantage is the possibility to estimate the status of the selected structural units: complexity and chain fragment. In consequence, a comparative analysis is possible.

## 5. Conclusions

Sequences of tetrapeptides representing a large difference of hydrophobicity in short and long polypeptide chains focused special attention on the protein structure prediction due to their differentiated status in micellar part of protein molecules. These sequences adopted different forms expressed by structural codes. In practice, by predicting the structural form of an early-stage intermediate, the procedure allowing for structural changes was introduced, since the specific forms of these tetrapeptides were able to determine the final status with respect to the micellar organization of final structures of proteins under consideration. Thus, an early step of folding for sequences under consideration shall be treated as influencing the structuralization already in an initial (early) step of folding.

## Figures and Tables

**Table 1:** Boundaries of intervals corresponding to structural codes (local maxima) expressed by the angle values in the parametric equation of the ellipse.

Code	From	To
A	0	50
B	51	85
C	86	110
D	111	150
E	151	193
F	194	225
G	226	359

**Table 2:** A set of tetrapeptide sequences showing the opposite distribution of structural codes for comparing the status compliant or noncompliant with the status expected by the fuzzy oil drop model.

A	C	D	E	F	G	H	I	K	L	N	P	Q
AENN AGHE AGYP AIIP AKNT ATGY AYGL AYPV	CVAS	DADE DAVP DFIV DFSK DIFL DIFT DIKF DKAG DKIC DLGS DLNP DPLD DPNG DPVP DVSG DYVF	EFYT EGYP EKFN EKKS EKNI ELYL ENVD EPKP ETPL EWVA EYEF EYIG	FGAD FGEP FIKN FQVV FRPG FVEV FVRL FVRN FGAD FGEP FIKN FQVV FRPG FVEV FVRL FVRN	GADE GAPE GFDI GHLK GNEV GNIN GNPV GSPI GSRL GTPA GTPN GTYI GWRL GYAV GYEI GYEV GYQL	HAEN HAKG HIVE HLDV HVAF	IAPV IAVD IEPI IEVQ IFFK IFNG IFTE IGSN IITY IKVN INIG INLH IPLK IPVA ISVW IVFD IVHR IVQF	KDFT KETF KLPA KLSL KLTK KLYS KLYY KVGI KYKL KYYA	LATP LDLQ LDSK LFDD LFLS LGEN LGLQ LHTN LHVH LIHG LNLR LPHV LPVY LRYD LSGH LSTP LSVG LTLY LVSY LWVE	NADS NEGA NGTR NKVL NPKV NVVC NVVG	PAVG PDDP PFIY PFLF PFVT PLKF PMMN PPPE PQGF PVLG	QIST QLEI QSLH
